# Different Contribution of Splanchnic Organs to Hyperlactatemia in Fecal Peritonitis and Cardiac Tamponade

**DOI:** 10.1155/2013/251084

**Published:** 2013-10-20

**Authors:** José Gorrasi, Anestis Eleftheriadis, Jukka Takala, Sebastian Brandt, Siamak Djafarzadeh, Lukas E. Bruegger, Hendrik Bracht, Stephan M. Jakob

**Affiliations:** ^1^Department of Intensive Care Medicine, University Hospital Bern (Inselspital), University of Bern, 3010 Bern, Switzerland; ^2^Department of Visceral Surgery and Medicine, University Hospital Bern (Inselspital), University of Bern, 3010 Bern, Switzerland

## Abstract

*Background*. Changes in hepatosplanchnic lactate exchange are likely to contribute to hyperlactatemia in sepsis. We hypothesized that septic and cardiogenic shock have different effects on hepatosplanchnic lactate exchange and its contribution to hyperlactatemia. *Materials and Methods*. 24 anesthetized pigs were randomized to fecal peritonitis (P), cardiac tamponade (CT), and to controls (*n* = 8 per group). Oxygen transport and lactate exchange were calculated during 24 hours. *Results*. While hepatic lactate influx increased in P and in CT, hepatic lactate uptake remained unchanged in P and decreased in CT. Hepatic lactate efflux contributed 20% (P) and 33% (CT), respectively, to whole body venous efflux. Despite maintained hepatic arterial blood flow, hepatic oxygen extraction did not increase in CT. *Conclusions*. Whole body venous lactate efflux is of similar magnitude in hyperdynamic sepsis and in cardiogenic shock. Although jejunal mucosal pCO_2_ gradients are increased, enhanced lactate production from other tissues is more relevant to the increased arterial lactate. Nevertheless, the liver fails to increase hepatic lactate extraction in response to rising hepatic lactate influx, despite maintained hepatic oxygen consumption. In cardiac tamponade, regional, extrasplanchnic lactate production is accompanied by hepatic failure to increase oxygen extraction and net hepatic lactate output, despite maintained hepatic arterial perfusion.

## 1. Introduction

Hyperlactatemia is a common finding in patients with sepsis and multiple organ failure and is associated with increased morbidity and mortality [1–3]. If such patients are well fluid resuscitated, systemic blood flow and oxygen delivery to the tissues are often high. Despite this, organ dysfunction and mild to moderate hyperlactatemia may persist.

In clinical practice, hyperlactatemia, especially in combination with metabolic acidosis, is commonly interpreted as sign of tissue hypoxia [4–7]. If sepsis is associated with hyperdynamic circulation, increased lactate production due to enhanced glycolytic metabolism, decreased oxygen extraction capability, and/or lactate clearance, and pyruvate dehydrogenase inactivation may offer alternative explanations for hyperlactatemia [8–12]. However, as a consequence of regional blood flow redistribution and metabolic compartmentalization, high systemic blood flow may not necessarily guarantee sufficient oxygen delivery to all tissues [13, 14]. In addition, sepsis often alters the function of organs which usually extract lactate, such as the liver and the kidneys. This may result in hyperlactatemia even in the presence of normal or only slightly increased lactate production. In contrast, in severe low cardiac output states, hyperlactatemia is likely to result from anaerobic metabolism and impaired lactate extraction due to generalized tissue hypoperfusion [15].

The aim of this study was to evaluate the contribution of hepatosplanchnic and extrahepatic lactate exchange to arterial hyperlactatemia in fecal peritonitis and cardiac tamponade. We hypothesized that the increase in arterial lactate concentration in fecal peritonitis is the result of a generalized increase in lactate production with maintained hepatic lactate uptake. 

## 2. Materials and Methods

### 2.1. Anesthesia, Monitoring, and Animal Preparation

The study was performed in accordance with the National Institutes of Health guidelines for the care and use of experimental animals and with the approval of the Animal Care Committee of the Canton of Berne, Switzerland. Here, we present data from a subset of animals from two previous publications, where effects of fluid resuscitation on mortality and organ function in sepsis [16] and oxygen transport and mitochondrial function in shock models [17] were investigated. 

24 pigs (median body weight 41 kg, range 38–44 kg) were deprived of food but not water 24 hrs before the experiment. They were premedicated with atropine 0.05 mg/kg of body weight, azaperone 6 xylazine 2 mg/kg, and ketamine 20 mg/kg intramuscularly, followed by cannulation of an ear vein and intravenous administration of 5–15 mg/kg pentobarbital and atropine 0.05 mg/kg followed by tracheal intubation. Anesthesia was maintained with pentobarbital 7 mg/kg hour and fentanyl 25 *μ*g/kg hour until the end of the operation and thereafter 3 *μ*g/kg hour. The animals were ventilated with a volume controlled ventilator with 5 cm of H_2_O end-expiratory pressure. FIO_2_ was adjusted to keep paO_2_ between 100 mm Hg and 150 mm Hg. Tidal volume was kept at 10 mL/kg, and the minute ventilation was adjusted to maintain paCO_2_ between 35 and 40 mm Hg. A pulmonary artery catheter and a femoral arterial catheter were inserted. Ringer's lactate was infused at 10 mL/kg hour. Additional fluid was administered if necessary to keep pulmonary capillary wedge pressure between 5 and 8 mm Hg. The aim was to keep the body temperature at 38 ± 0.5°C using and operating table heater and warmed fluids if necessary. 

### 2.2. Surgical Preparation

The carotid artery was exposed, and an ultrasound flow probe was placed around the artery (Transonic Systems, Ithaca, NY, USA). A large bore catheter for fluid administration was inserted into the femoral vein. The abdominal cavity was opened by a midline incision. A drainage catheter was inserted into the urinary bladder. The superior mesenteric, hepatic, splenic, and renal artery, the celiac trunk, and the portal vein were exposed, and ultrasound flow probes were placed around the vessels. Two fluid filled catheters were inserted into a mesenteric vein. The tip of the first catheter was placed into the portal vein, while the tip of the second catheter remained in the mesenteric vein. A catheter was inserted into the splenic vein and another catheter into the hepatic artery via the left gastric artery. A hepatic vein catheter was inserted, and the location of the tip was confirmed by ultrasound and oxygen vein saturation. The catheter was withdrawn 0.5 cm from the wedged position to allow measurement of hepatic venous pressure and saturation. A central venous catheter was inserted into the pericardial space via dissection of the left diaphragm. Before closing the laparotomy with clamps, two large bore tubes were placed with their tips in the peritoneal cavity to allow later insertion of autologous feces.

### 2.3. Hemodynamic Monitoring

Femoral and pulmonary arterial, central, hepatic, and portal venous blood pressures and pulmonary artery occlusion pressure were recorded with quartz pressure transducers and displayed continuously on a multimodular monitor (S/5 Compact Critical Care monitor, GE, Helsinki, Finland) and recorded. All pressure transducers were zeroed to the level of the heart. Cardiac output was measured by a thermodilution technique (mean value of three measurements, cardiac output module, S/5) and by using an esophageal Doppler probe (Cardio Q, Deltex Medical limitex, Chichester, UK). Central venous blood temperature (°C) was recorded from the thermistor in the pulmonary artery catheter and peripheral temperature from a thermistor placed between the toes. Regional blood flows were recorded by ultrasound transit time flow probes (Transonic Systems, Ithaca, NY, USA). Heart rate was measured from the ECG wich was also continuously monitored.

### 2.4. Experimental Protocol

After surgical preparation, 12 hours were allowed for hemodynamic stabilization. The animals were randomized into three different groups: peritonitis (*n* = 8), cardiac tamponade (*n* = 8), and controls (*n* = 8). Peritonitis was induced by instilling 1 g/kg of autologous feces suspended in warm 5% dextrose solution through a previously placed peritoneal drainage tube. Cardiac tamponade was induced by instilling warmed hydroxyethyl starch into the pericardial space with goals of cardiac output of 60 mL/kg/min during the first 6 hours, 50 mL/kg/min from 6 to 12 hours, and 40 mL/kg/min from 12 to 18 hours. Afterwards, cardiac output was not manipulated anymore. 

When clinical signs of hypovolemia developed in any group of the animals (rapidly decreasing blood pressure, increasing heart rate or urinary output <0.5 mL/kg/hr, and pulmonary artery occlusion pressure <5 mm Hg), additional aliquots of hydroxyethyl starch were infused. Glucose 50% was administered when blood glucose was <3.5 mmol/L. Animals surviving 24 hours were sacrificed with an overdose of intravenous potassium chloride.

### 2.5. Blood Measurements

Arterial hemoglobin and oxygen haemoglobin saturation were analyzed with an analyzer designed for porcine blood (OSM3, Radiometer, Copenhagen, Denmark). Blood gases were measured and temperature corrected in a blood gas analyzer (ABL 520, Radiometer, Copenhagen, Denmark). Arterial and venous blood lactate were measured with a lactate analyzer (YSI 2300 Stat Plus, Yellow Springs Instruments, CA, USA). 

### 2.6. Calculations and Statistics

Hepatic lactate influx (*μ*mol/kg/min) = (portal venous lactate × portal vein blood flow) + (arterial lactate × hepatic arterial blood flow). 

Hepatic lactate efflux (*μ*mol/kg/min) = hepatic venous lactate × (portal venous + hepatic arterial blood flow).

Hepatic lactate uptake (*μ*mol/kg/min) = hepatic lactate influx − hepatic lactate efflux.

Hepatic lactate extraction (%) = hepatic lactate efflux/hepatic lactate influx.

Other regional lactate exchanges: regional lactate influx − regional lactate efflux.

Area under arterial lactate concentration-time curve (mmol/L): sum of products of average of two consecutive lactate concentrations and time between the two measurements, divided by total time of experiment (mmol/L).

Whole body venous efflux: cardiac output × mixed venous lactate concentration. Extrahepatic organ lactate efflux: (cardiac output × mixed venous lactate concentration) − hepatic lactate efflux. 

For statistical analysis, the SPSS software package (SPSS version 14.1, SPSS Schweiz AG, Zurich, Switzerland) was used. In each group, nonparametric ANOVA for repeated measurements (Friedman test) was used to evaluate evolution over time. There was significant 24 hours mortality in peritonitis and tamponade groups. To ensure that the same number of measurements from each animal was available, only the last of the remaining measurements between 12 hours and 24 hours was used for analysis. For between-group comparison of variables with only one measurement, Kruskal Wallis test was employed. Statistical significance was set at *P* = 0.05. All results are presented as median and range.

## 3. Results

In both study groups, 4 animals died before 24 hours. 

### 3.1. Systemic Hemodynamics and Oxygen Transport

In both peritonitis and cardiac tamponade, shock ensued (Table 1). There was a transient increase in cardiac output in peritonitis and a decrease in cardiac tamponade. Systemic oxygen consumption was maintained in all groups (Table 1). 

### 3.2. Lactate Concentrations

Arterial and all regional lactate concentrations increased in peritonitis and in cardiac tamponade (Table 2). The area under the lactate concentration-time curve was 0.6 (0.5–1.1) mmol/L, 1.3 (0.9–1.7) mmol/L, and 1.3 (0.5–2.4) mmol/L for control, peritonitis, and cardiac tamponade groups, respectively (*P* = 0.005 for the comparison between all three groups). 

### 3.3. Lactate Exchange

**Figure 1:**
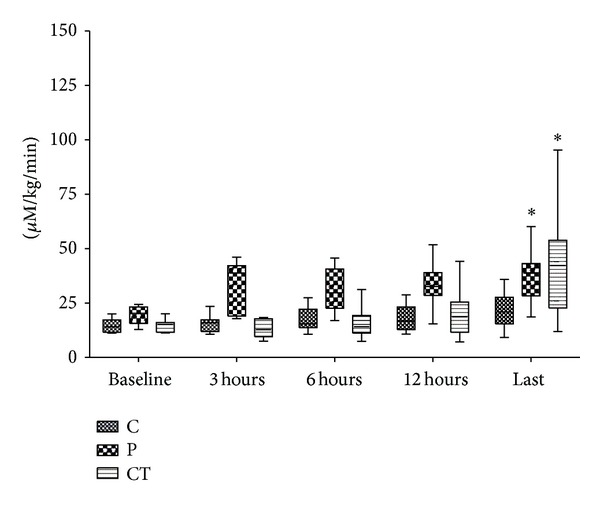
Hepatic lactate influx. *Friedmann test.

**Figure 2:**
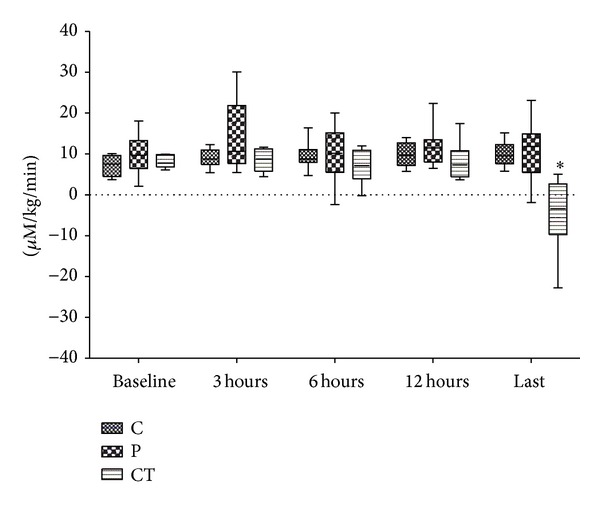
Hepatic lactate uptake. *Friedmann test.

Mesenteric lactate exchange—which was slightly negative at baseline (lactate release)—became early and transiently less negative in peritonitis (*P* = 0.031) and turned positive (lactate uptake) in the end in two thirds of the animals with tamponade (n.s.; ES-Figure  1 available online at http://dx.doi.org/10.1155/2013/251084). Renal lactate exchange was variable and did not change in either group (ES-Figure  2). 

**Figure 3:**
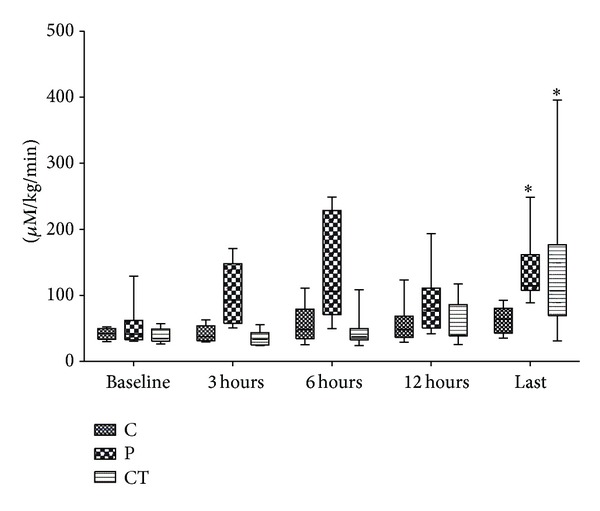
Extrahepatic organ lactate efflux. *Friedmann test.

Hepatic lactate influx increased early in peritonitis and late in cardiac tamponade (Figure 1; *P* = 0.001, both). Hepatic lactate uptake remained unchanged in control and peritonitis groups but turned to lactate release in all but two animals with cardiac tamponade (*P* = 0.006; Figure 2). Hepatic lactate efflux increased significantly in cardiac tamponade (*P* < 0.001) and tended to increase in peritonitis (*P* = 0.057; ES-Figure  3). Extrahepatic organ lactate efflux (Figure 3) and whole body venous lactate efflux (ES-Figure  4) increased both in peritonitis and in cardiac tamponade (all *P* < 0.012).

### 3.4. Regional Blood Flows and Oxygen Transport

In peritonitis, renal blood flow tended to decrease (*P* = 0.052), while all other regional flows were maintained (Table 3). In tamponade, celiac trunk and hepatic arterial blood flow were maintained, while all other regional flows decreased (Table 3). In peritonitis, all regional VO_2_ were maintained (ES-Table  1). In tamponade, oxygen extraction increased in all regions/organs but the liver. Only renal VO_2_ decreased significantly (ES-Table  1). 

### 3.5. Regional and Mucosal pCO_2_ Gradients

In peritonitis, mixed and hepatic venous-arterial and mucosal-arterial pCO_2_ gradients increased, while all other regional pCO_2_ gradients remained unchanged (ES-Table  2). In tamponade, jejunal mucosal and all regional venous-arterial pCO_2_ gradients increased (ES-Table  2).

## 4. Discussion

Our data indicate that the whole body venous lactate efflux as an estimate of hepatic and extrahepatic organ lactate efflux is of similar magnitude in the experimental models of severe peritonitis-induced sepsis and cardiac tamponade. However, changes in arterial lactate over time were due to different mechanisms. 

In peritonitis, whole body venous efflux increased early, mainly due to extrasplanchnic, extrarenal lactate production, and the liver lactate uptake was preserved. Unchanged regional pCO_2_ gradients and maintained regional oxygen consumption suggest that increased glycolysis rather than tissue hypoxia was the cause of increased lactate efflux. Despite a decrease in renal blood flow and an increase in local jejunal-mucosal pCO_2_ gradient, renal and mesenteric lactate exchange did not deteriorate, indicating that impaired perfusion in these regions was not critical either. 

In contrast, in cardiac tamponade, extrasplanchnic lactate production increased, and the hepatic lactate uptake reverted to net hepatic lactate efflux. The net hepatic lactate output was associated with a lack of increase in hepatic oxygen extraction. Although hepatic arterial perfusion was preserved, portal venous blood flow and oxygen content decreased markedly. We have previously demonstrated that hepatic lactate uptake can increase acutely by several folds, if hepatic perfusion is preserved [18, 19]. The failure of hepatic lactate uptake to increase in cardiac tamponade in the present study is likely explained by the decrease of hepatic perfusion and oxygen delivery below a critical level. We found previously in an acute short-term (150 minutes) cardiac tamponade model that hepatic lactate exchange decreased when hepatic oxygen delivery decreased to approximately 20% of control values (a mean of 0.3 mL/kg/min) [15]. In the present study, the sustained reduction of hepatic oxygen delivery to approximately 30% of the control values (a mean of 0.6 mL/kg/min) was associated with an average hepatic oxygen extraction of 0.75 and resulted in net hepatic lactate production. All regional pCO_2_ gradients increased, but the mesenteric (and femoral) lactate gradient did not increase. Accordingly, the decreased regional blood flows did not induce severe mesenteric tissue hypoxia. Hence, the net hepatic lactate production was a result of enhanced extra-mesenteric lactate efflux and hepatic lactate production. At high arterial lactate concentrations without mesenteric ischemia, the gut and other extrahepatic tissues may even take up lactate [19, 20]. In the present study, the arterial lactate concentrations remained only slightly or moderately increased, and the extrahepatic tissues continued to release lactate.

Despite the well-maintained hepatic perfusion in peritonitis, the liver lactate uptake did not increase. We have previously shown that hepatic lactate increases acutely during hyperlactatemia induced by lactate infusion [19] and in response to increased splanchnic lactate production during acute mesenteric ischemia [18], providing that the hepatic perfusion is preserved. The lack of increase in hepatic lactate uptake in peritonitis may be related to either the relatively modest increase in portal venous lactate, to sepsis-induced impairment of hepatic function unrelated to tissue perfusion, or to a combination of the two mechanisms. Regardless of the mechanism(s), the increased whole body lactate efflux in peritonitis was mainly due to increased extrasplanchnic lactate production. The lack of increase in regional pCO_2_-gradients suggests that the increased lactate efflux was not related to impaired regional perfusion. The etiology of hyperlactatemia in clinical sepsis without shock is multifactorial, but it is rarely associated with an increase in lactate to pyruvate ratio [21]. Accordingly, increased aerobic lactate production due to increased glycolysis and hypermetabolism or inhibition of pyruvate dehydrogenase activity and hepatic dysfunction may contribute. Indeed, it has been shown that stress-induced aerobic glycolysis in skeletal muscle occurs in many forms of shock including human and experimental septic shock and also hemorrhagic shock [22, 23]. Under these circumstances, hyperlactatemia (and increased pyruvate concentrations) is linked at least in part to epinephrine induced *β*_2_-stimulation of sarcolemmal Na^+^-K^+^-ATPase [24, 25]. 

In most patients with severe sepsis, the hepatosplanchnic region as a whole does not produce lactate [26]. This was also the case in peritonitis in the present study, where the hepatic-venous-arterial lactate gradient remained negative throughout the experiment.

Our study has important limitations. It has been shown in rabbits that fasting prevents or attenuates endotoxin-induced systemic and hepatic blood flow reduction, metabolic acidosis, and hyperglycemia which are observed in fed animals [27]. We cannot therefore exclude that feeding our animals until sepsis induction would have revealed a different metabolic (and maybe hemodynamic) response. Small doses of heparin infused to maintain intravenous catheter patency in our study may theoretically have interfered with the inflammatory profile in peritonitis animals [28]. In cardiac tamponade, the increased central venous pressures related to the model may have reduced the hepatic blood flow more than in cardiogenic shock caused by other mechanisms. This may have contributed to the net hepatic lactate production. The peritonitis model was fluid resuscitated throughout the study. In clinical sepsis, a variable delay in starting fluid resuscitation may modify both the etiology of lactate production and the lactate kinetics.

In conclusion, we found that venous lactate efflux from the systemic circulation was of a similar magnitude in peritonitis and in severe cardiogenic shock. In peritonitis, this was the consequence of an early increase in peripheral, extrasplanchnic, and extrarenal lactate production without signs of tissue hypoxia but impaired capability to increase hepatic lactate uptake. In cardiogenic shock, the increased venous lactate efflux was the result of increased net lactate release from both the periphery and from the liver. 

## Supplementary Material

The Supplementary Material contains 4 figures, indicating mesenteric (ES-Figure 1) and renal (ES-Figure 2) lactate exchanges, and hepatic (ES-Figure 3) and whole body vein lactate efflux (ES-Figure 4), as well as 2 tables indicating regional oxygen transport (ES-Table 1) and regional pCO2 gradients (ES-Table 2).

## Figures and Tables

**Table 1:** Systemic hemodynamics and oxygen transport.

		*N*	Baseline	3 hours	6 hours	12 hours	Last measurement	P*
Cardiac output (mL/kg/min)	Controls	8	89 (69–116)	91 (48–117)	98 (56–121)	92 (66–58)	108 (67–127)	0.344
	Peritonitis	8	90 (55–107)	87 (63–154)	114 (74–151)	82 (49–133)	102 (80–146)	0.060
	Tamponade	7	73 (54–101)	60 (38–76)	57 (53–76)	53 (47–81)	46 (38–62)	0.004

Central venous pressure (mmHg)	Controls	8	4 (2–6)	4 (2–7)	5 (3–8)	5 (3–7)	8 (5–9)	0.001
	Peritonitis	8	4 (0–5)	3 (1–5)	4 (2–6)	6 (3–9)	7 (4–10)	<0.001
	Tamponade	7	5 (3–9)	7 (4–9)	7 (6–10)	11 (7–15)	13 (12–16)	<0.001

Mean arterial pressure (mm Hg)	Controls	8	72 (61–82)	70 (52–91)	71 (56–89)	78 (64–81)	74 (50–91)	0.623
	Peritonitis	8	67 (61–92)	76 (54–92)	63 (54–88)	66 (49–89)	42 (34–90)	0.048
	Tamponade	7	80 (52–88)	66 (57–93)	64 (52–84)	56 (38–88)	36 (33–64)	<0.001

Systemic oxygen consumption (mL/kg/min)	Controls	8	4.76 (3.66–5.45)	4.42 (3.26–5.75)	4.70 (3.25–6.49)	4.78 (3.37– 11.31)	4.56 (3.22–5.45)	0.861
	Peritonitis	8	4.96 (2.50–5.98)	5.38 (4.20–7.86)	5.99 (4.39–6.88)	5.29 (3.19–6.14)	5.59 (3.55–6.43)	0.199
	Tamponade	7	4.17 (3.31–5.35)	4.07 (2.75–5.63)	3.87 (3.39–5.82)	4.35 (3.80–6.67)	4.17 (2.85–7.12)	0.966

Systemic oxygen extraction	Controls	8	0.43 (0.34–0.54)	0.41 (0.29–0.61)	0.47 (0.31–0.54)	0.46 (0.29–0.54)	0.42 0.28–0.51)	0.995
	Peritonitis	8	0.48 (0.36–0.59)	0.39 (0.31–0.42)	0.35 (0.29–0.50)	0.44 (0.33–0.66)	0.50 (0.32–0.66)	0.053
	Tamponade	7	0.48 (0.39–0.59)	0.54 (0.45–0.67)	0.59 (0.43–0.67)	0.62 (0.55–0.75)	0.82 (0.73–0.94)	<0.001

*Friedman test.

**Table 2:** Arterial, mixed and regional venous lactate concentrations.

		*N*	Baseline	3 hours	6 hours	12 hours	Last measurement	P*
Arterial lactate concentration	Controls	8	0.557 (0.439–0.999)	0.505 (0.160–0.766)	0.555 (0.451–1.880)	0.611 (0.453–0.863)	0.658 (0.450–0.993)	0.793
	Peritonitis	7	0.586 (0.391–0.960)	1.150 (0.679–2.270)	1.315 (0.614–2.200)	1.037 (0.721–2.010)	1.250 (0.972–2.720)	0.002
	Tamponade	7	0.586 (0.503–0.720)	0.600 (0.420–0.800)	0.650 (0.473–1.890)	0.935 (0.526–3.640)	3.560 (1.030–7.000)	0.001

Mixed venous lactate concentration	Controls	8	0.531 (0.443–0.754)	0.504 (0.443–0.729)	0.587 (0.470–1.220)	0.638 (0.507–0.808)	0.681 (0.461–1.050)	0.645
	Peritonitis	6	0.660 (0.417–1.390)	1.195 (0.757–2.120)	1.140 (0.797–2.370)	1.110 (0.714–2.090)	1.425 (0.884–2.750)	0.015
	Tamponade	7	0.600 (0.481–0.829)	0.619 (0.471–0.800)	0.753 (0.520–1.750)	0.972 (0.532–3.360)	3.510 (1.000–8.300)	0.001

Hepatic vein lactate concentration	Controls	8	0.275 (0.170–0.440)	0.270 (0.130–0.380)	0.325 (0.220–0.540)	0.310 (0.220–0.370)	0.375 (0.210–0.750)	0.502
	Peritonitis	7	0.420 (0.180–0.540)	0.860 (0.300–1.220)	0.840 (0.290–1.510)	0.745 (0.330–3.240)	0.910 (0.630–3.240)	0.010
	Tamponade	7	0.295 (0.200–0.894)	0.305 (0.180–0.643)	0.405 (0.250–1.170)	0.460 (0.200–3.090)	3.850 (0.640–8.400)	0.001

Portal vein lactate concentration	Controls	8	0.607 (0.493–0.792)	0.626 (0.521–0.815)	0.666 (0.545–1.280)	0.682 (0.599–0.859)	0.793 (0.535–1.030)	0.273
	Peritonitis	6	0.822 (0.557–0.954)	1.350 (1.210–2.010)	1.390 (0.817–2.250)	1.390 (0.970–2.040)	1.650 (1.170–3.100)	0.001
	Tamponade	6	0.699 (0.556–0.894)	0.731 (0.582–1.000)	0.800 (0.532–1.900)	1.110 (0.716–3.430)	3.400 (1.140–6.700)	0.004

Mesenteric vein lactate concentration	Controls	8	0.622 (0.427–0.807)	0.697 (0.504–0.777)	0.665 (0.553–1.250)	0.685 (0.590–0.823)	0.753 (0.591–1.030)	0.434
	Peritonitis	6	0.946 (0.586–1.380)	1.250 (0.882–1.840)	1.330 (0.816–3.510)	1.325 (1.020–2.360)	1.730 (1.020–2.970)	0.005
	Tamponade	8	0.729 (0.655–1.000)	0.846 (0.394–1.100)	0.780 (0.569–1.910)	1.140 (0.797–3.580)	2.715 (0.974–5.110)	0.002

Kidney vein lactate concentration	Controls	7	0.641 (0.505–1.000)	0.602 (0.468–0.965)	0.660 (0.504–1.690)	0.706 (0.541–0.948)	0.801 (0.393–1.510)	0.273
	Peritonitis	7	0.682 (0.390–1.290)	1.270 (0.903–2.050)	1.590 (0.974–2.510)	0.928 (0.762–3.670)	1.210 (1.140–2.660)	<0.001
	Tamponade	6	0.475 (0.409–0.919)	0.418 (0.372–0.800)	0.713 (0.418–1.860)	0.830 (0.401–2.830)	2.290 (0.634–7.000)	0.004

Femoral vein lactate concentration	Controls	8	0.919 (0.506–1.480)	0.765 (0.567–1.120)	0.830 (0.621–2.510)	0.852 (0.715–1.530)	1.245 (0.643–2.210)	0.141
	Peritonitis	5	0.834 (0.595–2.010)	1.690 (0.966–3.490)	1.935 (1.080–2.530)	1.720 (1.110–2.410)	1.980 (1.220–2.610)	0.014
	Tamponade	5	0.865 (0.549–2.380)	0.935 (0.769–1.050)	1.030 (0.783–3.180)	1.900 (1.310–3.960)	3.420 (1.520–6.600)	0.005

Spleen vein lactate concentration	Controls	8	0.702 (0.551–0.780)	0.758 (0.543–0.968)	0.759 (0.528–1.650)	0.699 (0.583–1.360)	0.742 (0.489–1.320)	0.393
	Peritonitis	4	0.718 (0.674–0.874)	1.720 (0.985–2.260)	1.650 (0.878–2.590)	1.645 (1.070–2.220)	1.860 (1.490–3.600)	0.007
	Tamponade	6	0.714 (0.569–1.000)	0.740 (0.708–1.140)	0.810 (0.640–1.960)	1.100 (0.636–3.770)	3.410 (1.550–8.300)	0.002

All concentrations are in mmol/L. *Friedman Test.

**Table 3:** Regional blood flows.

		*N*	Baseline	3 hours	6 hours	12 hours	Last measurement	P*
Superior mesenteric artery blood flow	Controls	8	17.64 (10.12–25.15)	19.01 (8.77–26.34)	17.68 (9.12–28.60)	18.74 (11.54–26.34)	19.68 (11.02–35.61)	0.772
	Peritonitis	8	16.06 (10.08–21.61)	14.32 (11.30–17.80)	16.12 (12.21–19.88)	15.23 (11.78–19.39)	16.37 (6.73–22.66)	0.897
	Tamponade	7	17.29 (12.21–26.59)	14.51 (12.59–18.25 )	15.69 (11.17–16.60)	13.40 (10.70–14.06)	11.90 (9.46–14.27 )	0.050

Portal vein blood flow	Controls	8	21.66 (13.00–26.37)	23.48 (12.31–29.77)	20.13 (12.51–33.94)	19.86 (14.21– 32.41)	24.53 (8.91–35.68)	0.974
	Peritonitis	8	21.91 (15.06–29.55)	19.55 (10.49–31.14)	20.66 (8.95–25.23)	17.71 (8.31–30.23)	18.23 (7.31–27.27)	0.628
	Tamponade	7	17.26 (14.50–30.57)	14.89 (10.68–20.75)	15.52 (9.93–19.41)	11.74 (6.40–16.18)	10.49 (7.67–15.21)	0.001

Renal artery blood flow	Controls	7	5.02 (3.11–8.10)	4.97 (2.75–8.24)	4.66 (2.53–9.22)	4.63 (3.30–10.74)	5.93 (2.42–9.87)	0.767
	Peritonitis	8	4.99 (3.65–8.06)	4.41 (3.51–7.00)	4.07 (2.72–5.50)	4.39 (0.51–6.29)	3.82 (0.51–7.65)	0.052
	Tamponade	7	5.05 (3.85–6.37)	3.85 (3.08–4.78)	3.75 (3.56–5.28)	3.13 (2.69–5.47)	1.53 (0.72–4.80)	0.001

Celiac trunk blood flow	Controls	8	6.32 (2.26–14.58)	5.73 (2.26–20.80)	7.40 (2.89–18.93)	8.93 (4.11–19.85)	9.10 (6.56–16.93)	0.008
	Peritonitis	8	7.70 (3.12–16.28)	8.20 (4.90–16.23)	8.53 (4.45–16.95)	11.61 (5.62–20.13)	10.00 (3.18–17.56)	0.701
	Tamponade	7	7.37 (3.03–11.89)	6.54 (4.70–13.14)	6.71 (3.95–11.47)	5.78 (3.79–10.48)	5.25 (3.07–10.62)	0.788

Hepatic artery blood flow	Controls	8	3.50 (0.71–5.27)	3.72 (0.63–8.45)	4.35 (0.87–7.87)	4.31 (1.33–7.59)	4.67 (1.95–6.03)	0.088
	Peritonitis	8	3.51 (1.69–11.91)	3.47 (1.27–10.75)	3.20 (1.01–11.13)	5.10 (1.16–13.06)	3.91 (1.43–11.36)	0.111
	Tamponade	7	2.48 (0.95–5.48)	3.06 (0.79–4.20)	3.21 (1.06–4.23)	2.45 (1.09–3.34)	1.58 (0.56–4.45)	0.613

Spleen artery blood flow	Controls	8	1.20 (0.46–1.74)	1.15 (0.39–1.92)	1.20 (0.47–2.14)	1.19 (0.53–2.74)	1.42 (0.52–3.14)	0.146
	Peritonitis	8	1.15 (0.71–1.55)	0.73 (0.36–1.47)	0.52 (0.22–2.04)	0.53 (0.21–2.29)	0.32 (0.02–2.09)	0.076
	Tamponade	7	1.03 (0.73–1.79)	0.82 (0.54–1.05)	0.87 (0.47–1.42)	0.67 (0.23–1.28)	0.38 (0.23–0.74)	<0.001

All flows are in mL/kg/min. *Friedman test.
