# Supplementary material for: Different Contribution of Splanchnic Organs to Hyperlactatemia in Fecal Peritonitis and Cardiac Tamponade
Source: Biomed Res Int. 2013 Oct 20;2013:251084. doi: 10.1155/2013/251084 (PMC3817825; doi:10.1155/2013/251084)
Supplement: Supplementary file 1 — The Supplementary Material contains 4 figures, indicating mesenteric (ES-Figure 1) and renal (ES-Figure 2) lactate exchanges, and hepatic (ES-Figure 3) and whole body vein lactate efflux (ES-Figure 4), as well as 2 tables indicating regional oxygen transport (ES-Table 1) and regional pCO2 gradients (ES-Table 2). [file 251084.f1.pdf]

Electronic Supplement

DIFFERENT CONTRIBUTION OF SPLANCHNIC ORGANS TO HYPERLACTATEMIA IN FECAL PERITONITIS AND CARDIAC TAMPONADE

José Gorrasi[1], Anestis Eleftheriadis[1], Jukka Takala[1], Sebastian Brandt[1], Siamak Djafarzadeh[1],

Lukas Bruegger[2], Hendrik Bracht[1], Stephan M. Jakob[1]

ES-Figure 1. Mesenteric lactate exchange.

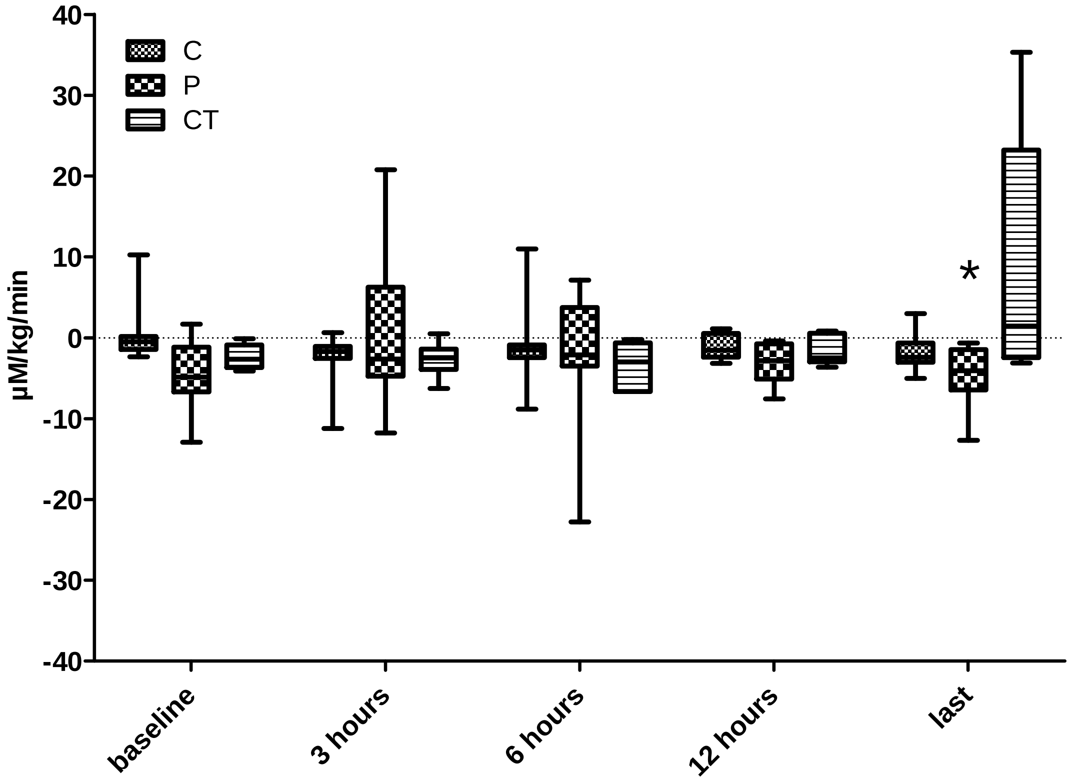

*Friedman Test.

ES-Figure 2. Renal lactate exchange

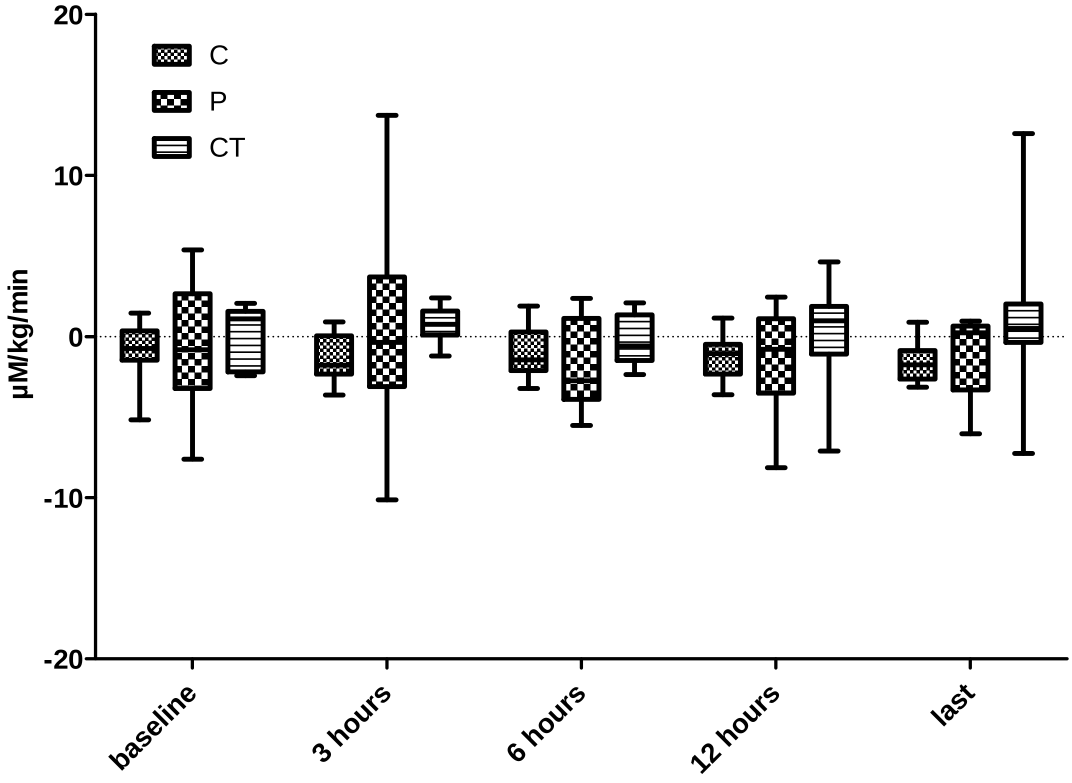

ES-Figure 3. Hepatic lactate efflux

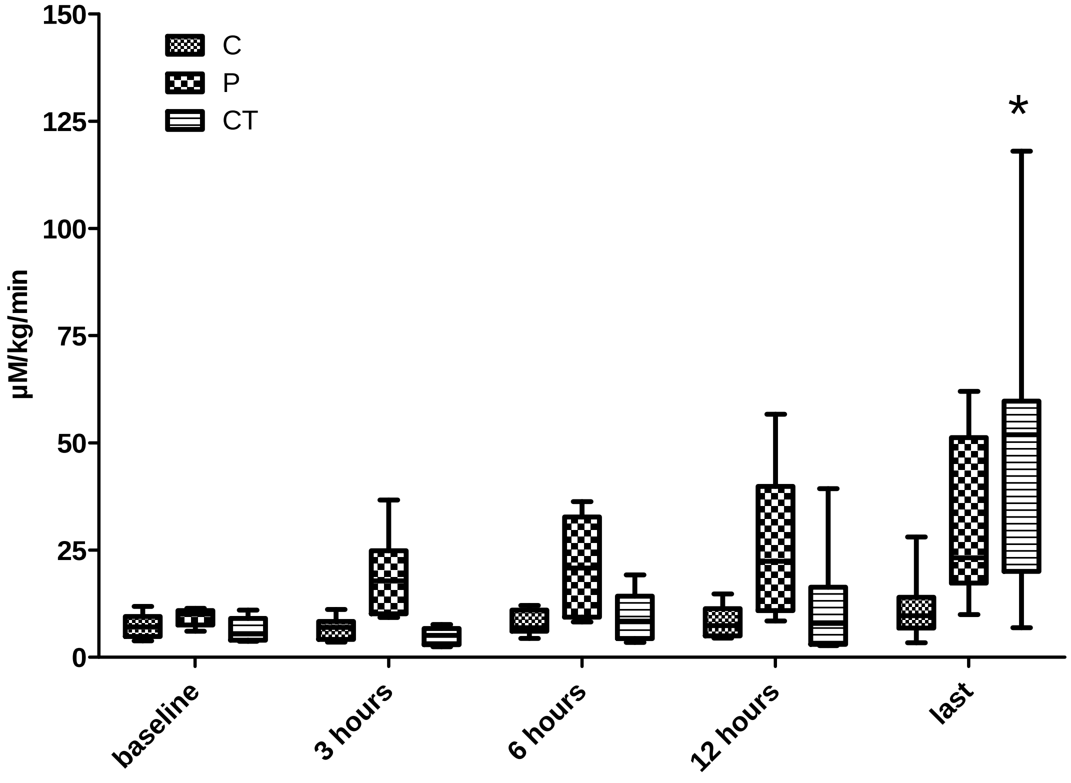

*Friedmann Test.

ES-Figure 4. Whole body vein lactate efflux (lung lactate influx)

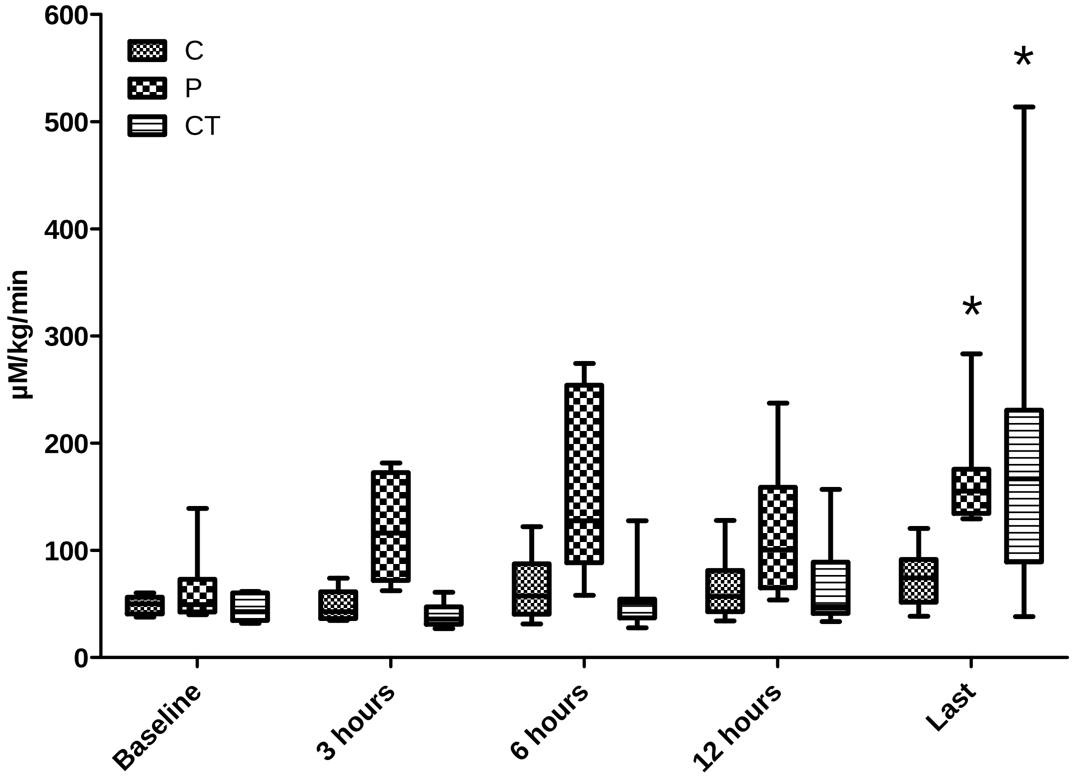

*Friedmann Test

ES-Table 1. Regional oxygen transport.

		N	Baseline	3 hours	6 hours	12 hours	Last measurement	p*
Hepatic oxygen consumption	Controls	8	0.86 (0.68 –1.18)	0.96 (0.78 – 1.66)	1.04 (0.69 – 1.38)	0.84 (0.65 – 1.40)	0.89 (0.37 – 1.34)	0.116
	Peritonitis	7	0.94 (0.43 – 1.23)	0.91 (0.23 – 1.22)	1.09 (0.32 – 1.35)	1.10 (0.38 – 1.50)	1.25 (0.38 – 1.97)	0.746
	Tamponade	7	0.82 (0.52 – 1.16)	0.79 (0.61 – 1.26)	0.74 (0.42 – 1.18)	0.65 (0.24 – 1.18)	0.47 (0.06 – 1.11)	0.284
Mesenteric oxygen consumption	Controls	8	0.57 (0.40 – 0.68)	0.63 (0.39 – 0.76)	0.63 (0.37 – 0.85)	0.62 (0.37– 1.03)	0.58 (0.33 – 1.02)	0.681
	Peritonitis	7	0.65 (0.46 – 0.76)	0.72 (0.42 – 1.04)	0.58 (0.53 – 1.13)	0.71 (0.38 – 0.88)	0.55 (0.17 – 0.93)	0.096
	Tamponade	7	0.61 (0.29 – 3.31)	0.68 (0.50 – 1.77)	0.68 (0.45 – 1.75)	0.69 (0.63 – 1.49)	0.76 (0.56 – 1.35)	0.849
Renal oxygen consumption	Controls	6	0.17 (0.09 – 0.30)	0.17 (0.08 – 0.28)	0.23 (0.13 – 0.32)	0.24 (0.12 – 0.45)	0.13 (0.10 – 0.34)	0.323
	Peritonitis	7	0.17 (0.14 – 0.38)	0.21 (0.16 – 0.28)	0.17 (0.13 – 0.21)	0.19 (0.15 – 0.23)	0.18 (0.09 – 0.52)	0.438
	Tamponade	7	0.16 (0.07 – 0.34)	0.14 (0.11 – 0.26)	0.16 (0.12 – 0.30)	0.16 (0.12 – 0.34)	0.09 (0.04 – 0.26)	0.041
Spleen oxygen consumption	Controls	8	0.02 (0.02 – 0.04)	0.03 (0.01 – 0.04)	0.03 (0.01 – 0.04)	0.02 (0.02 – 0.04)	0.02 (0.01 – 0.03)	0.925
	Peritonitis	6	0.03 (0.02 – 0.05)	0.03 (0.02 – 0.06)	0.03 (0.01 – 0.04)	0.03 (0.01 – 0.05)	0.03 (0.01 – 0.05)	0.687
	Tamponade	7	0.02 (0.02 – 0.03)	0.02 (0.02 – 0.03)	0.03 (0.02 – 0.05)	0.03 (0.01 – 0.07)	0.02 (0.02 – 0.05)	0.150
Hepatic oxygen extraction	Controls	8	0.41 (0.24 - 0.62)	0.57 (0.24 - 0.70)	0.49 (0.25 - 0.66)	0.38 (0.28 - 0.70)	0.42 (0.26 - 0.58)	0.681
	Peritonitis	7	0.36 (0.24 - 0.62)	0.29 (0.13 - 0.48)	0.36 (0.19 - 0.62)	0.32 (0.26 - 0.56)	0.56 (0.34 - 0.98)	0.022
	Tamponade	7	0.44 (0.31 - 0.52)	0.53 (0.39 - 0.80)	0.49 (0.24 - 0.77)	0.76 (0.19 - 0.79)	0.75 (0.33 - 0.88)	0.195
Mesenteric oxygen extraction	Controls	8	0.29 (0.17 - 0.35)	0.30 (0.17 - 0.46)	0.29 (0.16 - 0.46)	0.31 (0.13 - 0.40)	0.25 (0.16 - 0.50)	0.809
	Peritonitis	7	0.35	0.30	0.27	0.28	0.32	0.938

			(0.24 - 0.41)	(0.24 - 0.50)	(0.18 - 0.47)	(0.22 - 0.46)	(0.22 - 0.59)	
	Tamponade	7	0.32 (0.11 - 0.43)	0.37 (0.29 - 0.46)	0.40 (0.19 - 0.54)	0.41 (0.35 - 0.59)	0.61 (0.50 - 0.88)	0.002
Renal oxygen extraction	Controls	6	0.27 (0.18 - 0.42)	0.24 (0.19 - 0.46)	0.26 (0.23 - 0.52)	0.34 (0.24 - 0.50)	0.37 (0.17 - 0.45)	0.504
	Peritonitis	7	0.27 (0.18 - 0.50)	0.30 (0.21 - 0.38)	0.29 (0.24 - 0.43)	0.29 (0.24 - 0.42)	0.33 (0.28 - 0.58)	0.133
	Tamponade	7	0.26 (0.14 - 0.40)	0.33 (0.28 - 0.44)	0.36 (0.29 - 0.44)	0.42 (0.34 - 0.49)	0.53 (0.39 - 0.92)	0.001
Spleen oxygen extraction	Controls	8	0.20 (0.12 - 0.28)	0.17 (0.05 - 0.39)	0.18 (0.05 - 0.26)	0.14 (0.06 - 0.26)	0.13 (0.04 - 0.30)	0.681
	Peritonitis	6	0.26 (0.11- 0.43)	0.29 (0.12 - 0.56)	0.32 (0.10 - 0.43)	0.35 (0.09 - 0.43)	0.31 (0.13 - 0.60)	0.785
	Tamponade	7	0.21 (0.16 - 0.30)	0.27 (0.18 - 0.43)	0.32 (0.08 - 0.43)	0.43 (0.24 - 0.68)	0.70 (0.43 - 0.94)	0.001
Femoral oxygen extraction	Controls	8	0.51 (0.41 - 0.66)	0.46 (0.28 - 0.79)	0.40 (0.28 - 0.81)	0.45 (0.38 - 0.72)	0.52 (0.38 - 0.67)	0.483
	Peritonitis	7	0.54 (0.39 - 0.66)	0.36 (0.20 - 0.62)	0.40 (0.13 - 0.68)	0.58 (0.42 - 0.64)	0.56 (0.43 - 0.70)	0.323
	Tamponade	7	0.55 (0.41 - 0.75)	0.67 (0.52 - 0.74)	0.63 (0.45 - 0.76)	0.72 (0.56 - 0.76)	0.84 (0.76 - 0.94)	0.010

All oxygen consumption rates are in ml/kg/min. *Friedman Test.

ES-Table 2. Regional pCO_2 gradients.

		N	Baseline	3 hours	6 hours	12 hours	Last measurement	p*
Mixed venous-arterial PCO_2 gradient	Controls	8	8.0 (6.5 – 9.2)	6.8 (5.3– 10.5)	8.0 (1.7 – 12.1)	8.1 (5.3 – 10.6)	8.4 (4.6 – 12.9)	0.910
	Peritonitis	7	7.7 (0.9 – 10.3)	9.7 (6.1 – 12.5)	10.0 (7.0 – 23.5)	10.9 (7.2 – 11.9)	10.9 (8.5 – 17.4)	0.017
	Tamponade	7	8.7 (7.1 – 10.4)	9.7 (6.5 – 14.0)	10.3 (8.4 – 13.7)	14.2 (10.4 – 16.7)	20.9 (15.2 – 64.4)	<0.001
Hepatic venous-arterial PCO_2 gradient	Controls	8	7.7 (6.1 – 10.8)	7.5 (4.1 – 12.4)	7.6 (4.6 – 13.4)	7.8 (3.2 – 12.0)	8.5 (5.0 – 16.9)	0.897
	Peritonitis	6	8.5 (-1.50 – 14.5)	10.7 (4.3 – 14.8)	9.9 (7.2 – 23.7)	10.7 (7.6– 16.4)	12.3 (6.7 – 22.2)	0.004
	Tamponade	7	7.7 (0.2 – 11.1)	10.1 (4.00 – 15.9)	10.6 (7.5 – 13.5)	14.9 (10.4– 20.2)	22.8 (14.0 – 72.0)	<0.001
Portal venous-arterial PCO_2 gradient	Controls	8	7.4 (5.6 – 9.2)	8.3 (4.2 – 10.2)	7.3 (1.4– 12.0)	7.6 (4.0 – 9.70)	8.9 (2.6 – 10.3)	0.775
	Peritonitis	7	8.5 (5.0 – 13.2)	10.4 (4.3 – 13.2)	10.5 (6.3 – 12.8)	9.6 (5.8 – 15.3)	11.1 (6.1 – 19.3)	0.909
	Tamponade	6	7.6 (4.7 – 9.0)	9.1 (7.9– 11.3)	8.4 (7.1 – 12.3)	13.2 (10.6 – 16.7)	17.9 (13.6 – 74.4)	<0.001
Mesentric venous-arterial PCO_2 gradient	Controls	8	7.0 (4.7 – 10.2)	9.0 (4.0 – 13.6)	7.9 (3.8 – 12.4)	8.2 (4.7 – 11.4)	7.1 (4.2– 13.4)	0.784
	Peritonitis	7	9.2 (6.8 – 15.3)	9.5 (8.0 – 13.1)	12.5 (6.2 – 22.8)	10.2 (8.2 – 19.9)	11.0 (7.5 – 20.9)	0.932
	Tamponade	6	9.2 (7.8 – 14.1)	11.5 (9.0 – 13.3)	10.2 (5.9 – 17.5)	15.4 (10.3 – 19.5)	20.1 (16.8 – 46.0)	0.002
Renal venous-arterial PCO_2 gradient	Controls	7	5.4 (2.2– 9.4)	4.8 (2.3– 7.1)	4.1 (-0.5– 11.5)	5.8 (2.8– 11.7)	5.3 (2.2– 9.8)	0.256
	Peritonitis	7	4.2 (2.3 – 11.1)	6.4 (3.4 – 10.7)	6.6 (3.7 – 17.9)	6.0 (4.5 – 10.6)	5.2 (3.4 – 14.9)	0.300
	Tamponade	7	4.0 (1.8 – 8.0)	3.8 (1.0 – 10.6)	5.0 (3.5 – 9.8)	7.0 (5.0 – 11.6)	8.2 (4.3 – 71.2)	0.001
Femoral venous-arterial PCO_2 gradient	Controls	8	10.8 (8.6 – 12.8)	9.6 (7.6 – 13.8)	10.4 (3.2 – 15.6)	11.1 (4.8 – 16.9)	10.9 (6.5 – 25.5)	0.827
	Peritonitis	6	11.8	10.7	15.5	15.2	15.6	0.121

			(6.3 – 13.4)	(-0.4 – 18.7)	(4.0 – 24.5)	(9.8 – 16.4)	(10.6 – 23.7)	
	Tamponade	5	11.8 (8.9 – 16.1)	13.7 (11.8 – 17.5)	13.7 (11.6 – 19.2)	17.9 (15.6 – 20.1)	25.0 (19.2 – 69.50)	0.002
Spleen venous-arterial PCO_2 gradient	Controls	8	4.5 (3.7 – 7.9)	4.6 (1.6 – 9.4)	5.0 (-2.0 – 8.7)	4.2 (1.6 – 7.2)	4.7 (1.2 – 11.2)	0.914
	Peritonitis	5	6.5 (3.0 - 8.3)	11.6 (3.9 - 15.0)	13.6 (4.3 – 22.4)	11.6 (3.9 - 18.5)	6.5 (3.0 - 24.3)	0.316
	Tamponade	6	5.0 (3.4 - 8.6)	7.2 (4.4 - 9.6)	8.2 (3.0 - 11.7)	12.0 (7.1 - 18.6)	16.1 (7.3 – 89.9)	0.010
Jejunal mucosal-arterial PCO_2 gradient	Controls	7	19.5 (-1.8 - 27.2)	14.8 (8.8 - 23.7)	21.5 (8.0 - 22.9)	21.1 (7.4 - 70.6)	30.2 (20.3 - 49.6)	0.017
	Peritonitis	7	26.6 (15.4 - 72.1)	31.5 (14.1 - 69.5)	33.8 (12.7 - 56.3)	50.4 (24.4 - 112.5)	67.4 (12.5 - 112.5)	0.005
	Tamponade	7	25.2 (15.4 - 72.1)	34.0 (14.1 - 69.5)	35.6 (12.7 - 56.3)	71.6 (24.4 - 112.5)	75.0 (12.5 - 112.5)	0.014

All pCO_2 gradients are in mmHg. *Friedman Test.
